# Microbial and Immune Landscape of Malignant Ascites: Insights from Gut, Bladder, and Ascitic Fluid Analyses

**DOI:** 10.3390/cancers17081280

**Published:** 2025-04-10

**Authors:** Jina Yun, Ju-Sun Song, Jeong-Ju Yoo, Solbi Kweon, Yoon-Young Choi, Daero Lim, Jung-Cheol Kuk, Hyun-Jung Kim, Seong-Kyu Park

**Affiliations:** 1 Division of Hematology-Oncology, Department of Medicine, Soonchunhyang University Bucheon Hospital, Soonchunhyang University College of Medicine, Bucheon 14584, Republic of Korea; 68514@schmc.ac.kr (H.-J.K.); skpark@schmc.ac.kr (S.-K.P.); 2 GC Genome, Department of Laboratory Medicine, Green Cross Laboratories, Seoul 16924, Republic of Korea; jssong@gccorp.com (J.-S.S.); solbik@gccorp.com (S.K.); 3 Division of Hepatology, Department of Medicine, Soonchunhyang University Bucheon Hospital, Bucheon 14584, Republic of Korea; puby17@schmc.ac.kr; 4 Department of Surgery, Soonchunhyang University Bucheon Hospital, Soonchunhyang University College of Medicine, Bucheon 14584, Republic of Korea; biosurgeon@schmc.ac.kr (Y.-Y.C.); limdaero@schmc.ac.kr (D.L.); kjc1110@schmc.ac.kr (J.-C.K.)

**Keywords:** malignant ascites, microbiome, 16S rRNA, gut, bladder, immunosuppressive environment

## Abstract

Background/Objectives: Malignant ascites frequently arises in advanced cancers with peritoneal metastasis and is associated with poor outcomes. Known mechanisms include lymphatic obstruction by tumor cells, increased vascular permeability, and sodium retention via the renin–angiotensin–aldosterone system; however, the pathogenesis remains not fully understood. We investigated whether gut and bladder microbiomes correlate with malignant ascites development or progression and whether the immune microenvironment in ascitic fluid is altered. Methods: We enrolled 66 histologically confirmed cancer patients, dividing them into malignant ascites (n = 20) and non-ascites (n = 46) groups. Stool, urine, and ascitic fluid samples were analyzed using 16S rRNA next-generation sequencing. Immune cell subsets in ascitic fluid were characterized using flow cytometry. Results: In 19 of the 20 malignant ascites samples, the bacterial load was too low for reliable 16S rRNA sequencing, suggesting that malignant ascites is largely sterile. The overall gut microbiome diversity did not differ significantly by ascites status, although a trend emerged in patients with peritoneal metastasis, including the enrichment of class Clostridia and Gammaproteobacteria. Bladder microbiome analysis also showed no significant differences in ascites or metastasis status. Flow cytometry revealed reduced T-cell (CD3+, CD4+, CD8+) and NK cell (CD56+) populations compared to data from cirrhotic ascites. Conclusions: Malignant ascites exhibit minimal bacterial biomass, making comprehensive microbiome analysis challenging. Although no major global changes were noted in gut and bladder microbiomes, specific taxa were linked to peritoneal metastasis. These findings highlight an immunosuppressive ascitic environment and suggest that larger-scale or multi-omics approaches may help elucidate the role of microbiota in malignant ascites.

## Simple Summary

Malignant ascites, a fluid buildup in the abdomen due to advanced cancer, is associated with poor prognosis; yet, its underlying causes remain unclear. This study explores whether microbes from the gut and bladder contribute to malignant ascites and examines the immune environment within the fluid. Using genetic sequencing and immune cell analysis, we found that malignant ascites contains very few bacteria, making it difficult to assess microbial influences. While overall gut and bladder microbiomes showed no significant changes, specific bacterial groups were linked to peritoneal metastasis. Additionally, immune cell populations in malignant ascites suggested a suppressed immune response. These findings highlight the complexity of malignant ascites and suggest that larger studies using advanced techniques may be needed to better understand its development and potential treatment strategies.

## 1. Introduction

Malignant ascites, characterized by the presence of malignant cells in ascitic fluid cytology, commonly arises in patients with advanced cancer and peritoneal metastases, accounting for approximately 53.3% of cases associated with malignancy-related ascites [1]. The development of malignant ascites typically signifies diminished responsiveness to anticancer therapies and correlates with a poor prognosis, with a median survival time of only one to four months [2].

The pathogenesis of malignant ascites is multifactorial and not fully understood. Although lymphatic obstruction due to cancer cells contributes to its development, this alone does not account for the complete mechanism of ascites formation. Additional mechanisms, such as RAAS-mediated sodium retention secondary to reduced circulating blood volume, and hormonally driven changes in vascular permeability—mediated by factors including VEGF, IL-2, and TNF-alpha—are also implicated [3]. Despite advances in understanding these processes, the full pathophysiology remains elusive, underscoring the need for further exploration to identify new therapeutic targets.

Advances in high-sensitivity 16S rRNA next-generation sequencing for bacterial detection have heightened interest in the microbiome’s role across various diseases, particularly cancer [4,5]. Notably, the most intensive research is currently focused on colon cancer, revealing that the intestinal microbial community undergoes various changes throughout the development and progression of the disease. This microbial community significantly influences epithelial–mesenchymal transition (EMT) and the gut–vascular barrier, and it is recognized as a key factor that impacts the tumor microenvironment by altering the surrounding immune landscape and releasing signaling metabolites [6]. While the gut microbiome has garnered the most attention in cancer research, recent studies suggest that the bladder also harbors a distinct microbial community that can influence local inflammation and host immunity [7]. However, its potential role in malignant ascites remains unclear. Considering that malignant ascites often present an immunosuppressive milieu, understanding how the local microbiome interacts with immune cells in the peritoneal cavity could unveil novel therapeutic targets or diagnostic markers [8].

Given these insights, we hypothesized that the gut and bladder microbiomes—two anatomically adjacent yet distinct microbial niches—may contribute to or reflect the immunologic milieu of malignant ascites. Using 16S rRNA sequencing and flow cytometry, we analyzed stool (gut), urine (bladder), and ascitic fluid samples, aiming to elucidate potential microbial and immunological interplays in malignant ascites.

## 2. Materials and Methods

### 2.1. Patients and Study Protocol

Patients were divided into two groups: (1) those with malignant ascites (ascites detected on imaging and/or cytology confirming malignant cells) and (2) those without ascites. Patients with positive bacterial cultures of ascitic fluid or urine, antibiotic use within two weeks before enrollment, or inability to obtain sterile ascitic fluid or urine samples were excluded. This study was approved by the Institutional Review Board of Soonchunhyang University Bucheon Hospital (IRB No. 2021-04-034, date of approval: 16 June 2021), and all participants provided written informed consent.

Ascitic fluid was obtained by paracentesis and analyzed for cell counts, culture, cytopathology, and flow cytometry (CD3, CD4, CD8, CD19, CD56, HLA-DR). Flow cytometry was performed using Beckman Coulter NAVIOS (Beckman Coulter, Brea, CA, USA) and analyzed according to the manufacturer’s protocol. Urine was collected via transurethral catheterization under sterile conditions and subjected to routine urinalysis, Gram stain, culture, and 16S rRNA analysis. Stool was self-collected using a commercial collection kit (NBgene-GUT kit; Noble Biosciences, Hwaseong-si, Republic of Korea), shipped within 72 h, and immediately stored at −80 °C until analysis.

### 2.2. DNA Extraction and 16S rDNA Sequencing

This protocol, adapted from previous studies focusing on malignant and cirrhotic ascites, was specifically tailored to address malignant ascites in this study [9]. Malignant ascitic fluid samples (~25 mL each) were transported under refrigeration (4 °C) to the genetic laboratory, and DNA extraction began within 24 h. To minimize contamination, all samples were processed in a laminar flow hood, and negative controls were included. Based on previously published methods, malignant cells, which are abundant in malignant ascites, were removed to prevent interference with subsequent PCR amplification targeting bacterial 16S rRNA genes. A two-step differential centrifugation protocol was employed to concentrate bacterial pellets. The initial centrifugation was performed at 2770 rcf for 10 min to separate mammalian cells. The supernatants were then removed, and subsequent centrifugation was conducted at 4000 rcf for 10 min. From the resulting pellet, 300 μL of the specimen was collected and processed for DNA extraction using the MagMAX™ Microbiome Ultra Nucleic Acid Isolation Kit (ThermoFisher Scientific, Waltham, MA, USA) in accordance with the manufacturer’s instructions. To verify the absence of contamination, negative controls were processed simultaneously.

Urine samples (25 mL) were collected and stored in boric acid at 4 °C, then transported to the genetic laboratory within one day. Following centrifugation at 3300× *g* for 30 min, the urinary pellet was subjected to DNA extraction using the MagMAX™ Microbiome Ultra Nucleic Acid Isolation Kit (ThermoFisher Scientific, Waltham, MA, USA). A negative DNA extraction control, consisting of reagents without urine samples, was processed to verify the absence of contamination. This method was adapted from previously published protocols with slight modifications [10].

Stool samples were self-collected with a stool collection kit containing preservatives (NBgene-GUT kit; Noble Biosciences, Hwaseong-si, Republic of Korea). All samples were received within three days of collection. Upon arrival, DNA was extracted using the Chemagic DNA Stool Kit (PerkinElmer, Waltham, MA, USA), incorporating a modified bead-beating pretreatment step (1 min, FastPrep-24 homogenizer, MP Biomedicals, Irvine, CA, USA).

The V4 region of the 16S rRNA gene was amplified and sequenced, following a protocol adapted from previously published studies. Extracted DNA was utilized to construct a 16S rRNA gene library using the NEXTflex 16S V4 Amplicon-Seq kit (BioO Scientific, Austin, TX, USA). The library quality was evaluated with the 4200 Tape Station System (Agilent Technologies, Santa Clara, CA, USA). Paired-end sequencing was performed on a MiSeq 2000 platform with the MiSeq Reagent Kit v2 nano, according to the manufacturer’s instructions (Illumina, San Diego, CA, USA). To ensure high-quality sequencing data, 12% PhiX DNA (Illumina, San Diego, CA, USA) was included in the sequencing runs.

### 2.3. Bioinformatic Analysis of the Gut Microbiome and Statistical Analysis

We used QIIME2 (version 2023.2) to analyze the 16S sequence data [11]. Demultiplexed and primer-trimmed data were quality-filtered and denoised using the DADA2 (version 1.16) plugin [12]. Amplicon sequence variants with fewer than 10 reads or those present in only a single sample were removed, and taxonomy was assigned to each amplicon sequence variant using the naive Bayes machine-learning taxonomy classifiers in the q2-feature-classifier [13] trained against the NCBI Reference Sequence (RefSeq) database.

Alpha-diversity was determined using the Shannon index, and beta-diversity was calculated using the Bray–Curtis distance in QIIME2 software. To visualize the microbial community, principal coordinates analysis plots were generated using an R script. A Linear discriminant analysis effect size (LEfSe) approach was employed to identify potential microbial markers associated with ascites status (default LDA > 2) [14].

Differences in the alpha diversity between the two groups and three groups were compared using the Wilcoxon test and the Kruskal–Wallis test, respectively. PERMANOVA using the “adonis” command in the vegan package (version 2.6.4) in R (10,000 simulations) [15] was used for the beta-diversity comparison.

### 2.4. Ethics Statement

The study was approved by the institutional review board (IRB) of Soonchunhyang University Bucheon Hospital (IRB No. 2021-04-034). Written consent was obtained from all enrolled participants.

## 3. Results

### 3.1. Baseline Characteristics of the Study Population

**Figure 1:**
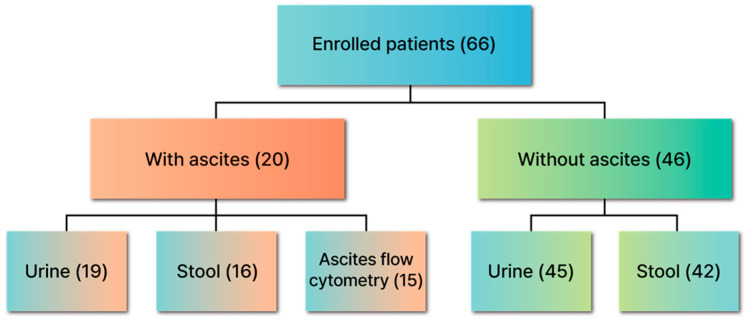
Flow diagram of patient enrollment and sample collection. A total of 66 patients were enrolled, of whom 20 presented with malignant ascites and 46 did not. Among those with ascites, 19 provided urine samples, 16 provided stool samples, and 15 underwent flow cytometric analysis of ascitic fluid. Among those without ascites, 45 provided urine samples and 42 provided stool samples.

A total of 66 patients were enrolled in this study (Table 1 for baseline demographics; Figure 1 for enrollment flow). The mean age was 64.79 ± 10.84 years (range: 31–87), with a male-to-female ratio of 1:1 (33 males, 50%). Colorectal cancer was the most common malignancy (n = 48, 72.7%), followed by ovarian cancer (n = 10, 15.2%), gastric cancer (n = 6, 9.1%), and others (n = 2, 3.0%). By clinical stage, 18 (27.3%) had stage I/II, 19 (28.8%) had stage III, and 29 (43.9%) had stage IV.

Among all participants, 20 (30.3%) presented with ascites, whereas 46 (69.7%) did not. Of those with ascites, pathologically confirmed peritoneal metastasis was observed in twelve (60.0%), atypical cells in six (30.0%), and no malignant findings in two (10.0%). As shown in Figure 1, of the 20 patients with ascites, 19 (95.0%) provided urine samples, 16 (80.0%) provided stool samples, and 15 (75.0%) underwent ascites flow cytometry. Of the 46 patients without ascites, 45 (97.8%) provided urine samples and 42 (91.3%) provided stool samples.

### 3.2. Malignant Ascites Typically Shows Minimal Bacterial Load

Among the 20 ascitic fluid samples, 19 yielded insufficient DNA for reliable 16S rRNA amplification, implying a nearly sterile environment (Table 2). One sample grew *Enterococcus faecalis* in both culture and sequencing. WBC and PMN counts varied widely (WBC 140–6768 cells/μL; PMN 1–5617 cells/μL), showing no clear correlation with bacterial load. Ascitic fluid CEA levels ranged from 0.33 to 495,370 ng/mL, reflecting diverse cancer types and disease burdens.

### 3.3. Gut Microbiome Shifts: Higher Diversity in Stage IV Colorectal Cancer

#### 3.3.1. Overview and Subgroup Comparisons

**Figure 2:**
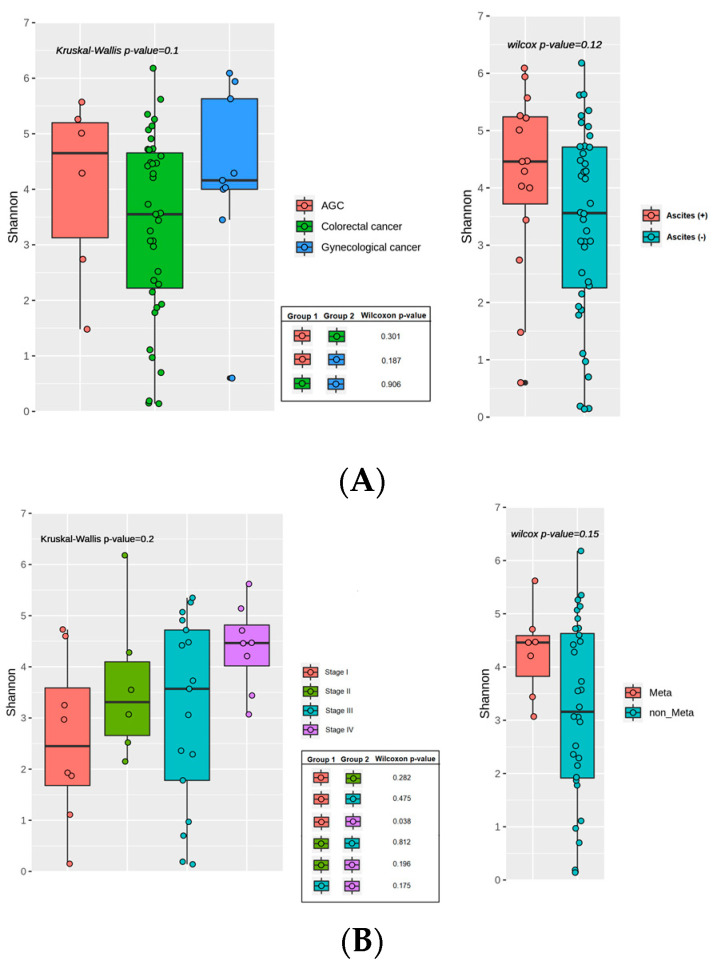
Alpha diversity of the gut microbiome (Shannon index): (**A**) Comparison by cancer type (colorectal, gastric, and gynecological) and ascites status (Ascites+ vs. Ascites−). (**B**) Subgroup analysis in colorectal cancer by clinical stage (I–IV) and peritoneal metastasis (Meta vs. non-Meta). The Kruskal–Wallis test showed no global difference among stages (*p* = 0.20), but pairwise comparisons revealed significantly higher diversity in stage IV vs. stage I (*p* = 0.04).

Stool samples from 55 patients were available for microbiome analysis. All 55 patients were compared according to types of cancer (colorectal vs. gastric vs. gynecological) and the presence or absence of ascites (Figure 2A). Additionally, subgroup analyses in colorectal cancer patients (n = 40) were performed by clinical stage (I/II/III/IV) and by the presence or absence of peritoneal metastases (Figure 2B).

#### 3.3.2. Alpha Diversity: Stage IV vs. Stage I Colorectal Cancer

In the overall cohort, alpha diversity (Shannon index) did not differ significantly by cancer type (*p* = 0.10) or ascites status (*p* = 0.12). Among the 40 colorectal cancer patients, there was no significant difference across stages I/II/III/IV (*p* = 0.20, Kruskal–Wallis), although stage IV showed higher diversity than stage I (*p* = 0.04), though the small sample size warrants cautious interpretation. Apart from this comparison, no other subgroup analyses, including the presence vs. absence of peritoneal metastasis, demonstrated significant differences in alpha diversity in the colorectal cancer cohort.

#### 3.3.3. Beta Diversity: Subtle Changes Without Significant Clustering

Beta diversity based on the Bray–Curtis distance (visualized via PCoA) did not show significant global clustering differences by stage, peritoneal metastasis, cancer type, or ascites status (PERMANOVA *p* > 0.05 for all comparisons). Nevertheless, there was a subtle compositional shift between patients with (green) vs. without (orange) peritoneal metastasis, though it did not reach statistical significance (Figure S1). Specifically, PCoA revealed partial spatial separation along PC1 (24.35% of variance explained), with patients harboring peritoneal metastases tending to cluster toward the negative axis. However, the overall difference was insignificant (PERMANOVA *p* = 0.59), and 95% confidence ellipses showed substantial overlap. These findings suggest that, although a mild compositional shift may exist, it does not reach statistical significance under the current sample size and distance metric.

#### 3.3.4. LEfSe Reveals Clostridia and Gammaproteobacteria Enrichment in Metastatic Cases

**Figure 3:**
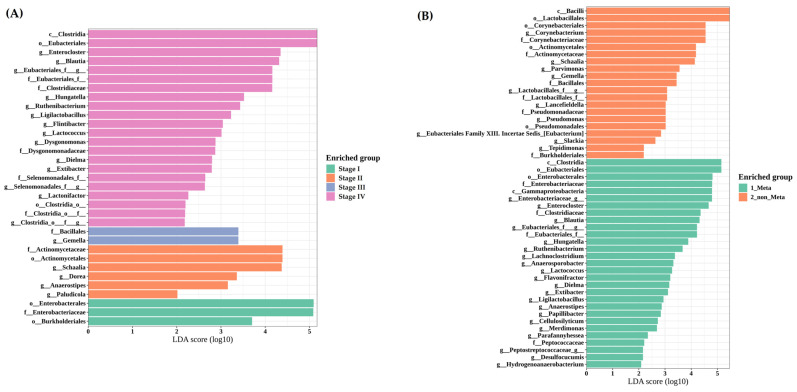
LEfSe analysis of the gut microbiome by clinical stage and peritoneal metastasis status: (**A**) Differentially enriched taxa among stages I, II, III, and IV in colorectal cancer. (**B**) Differences between metastatic (Meta) and non-metastatic (non-Meta) cases. The LDA scores are shown on the left.

Taxonomic profiling (LEfSe analyses) indicated that among stage IV colorectal cancer patients, members of the class Clostridia were relatively enriched. In those with peritoneal metastases, Clostridia and Gammaproteobacteria were more abundant (Figure 3A), whereas patients without peritoneal metastases showed enrichment of class Bacilli (Figure 3B). No statistically significant differences in bacterial composition were found by cancer type (colorectal vs. gastric vs. gynecological). To improve clarity and facilitate interpretation, taxa abundance bar plots were added in Supplementary Figure S2, enabling direct comparison with the LEfSe outputs shown in Figure 3.

### 3.4. Urine Microbiome Remains Largely Unchanged Across Clinical Groups

**Figure 4:**
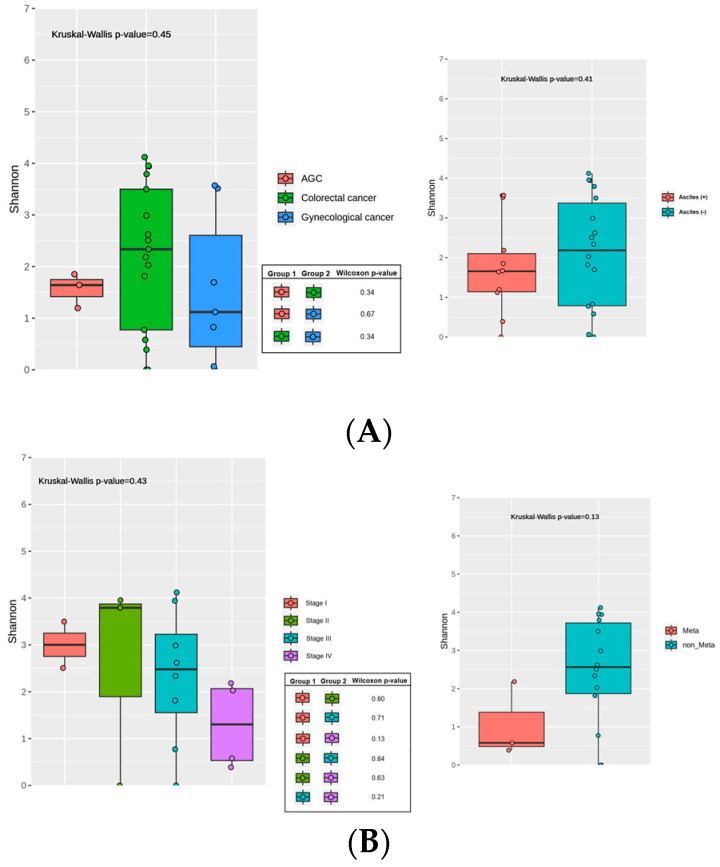
Alpha diversity (Shannon index) of the urine microbiome across different clinical groups: (**A**) Comparisons among gastric, colorectal, and gynecological cancers, and between patients with (Ascites+) and without (Ascites−) malignant ascites. (**B**) In colorectal cancer patients, alpha diversity across stages I–IV and by peritoneal metastasis (Meta vs. non-Meta). Statistical significance was determined using Wilcoxon rank-sum or Kruskal–Wallis tests; *p*-values are indicated within each plot.

Of the 64 initial urine samples collected, 55 had corresponding stool samples available for concurrent analysis. However, twenty-one were excluded due to insufficient bacterial load, four failed during the initial QIIME pipeline, and two were lost. Consequently, 28 samples were successfully analyzed for the urinary microbiome. All 28 patients were compared according to their cancer types (colorectal vs. gastric vs. gynecological) and the presence or absence of ascites (Figure 4A). Additionally, subgroup analyses in colorectal cancer patients (n = 17) were performed by clinical stage (I/II/III/IV) and the presence or absence of peritoneal metastases (Figure 4B).

#### 3.4.1. Alpha Diversity: No Notable Variation by Cancer Type, Stage, or Ascites

In the overall cohort of 28 patients, alpha diversity (Shannon index at the species level) did not differ significantly by cancer type (colorectal vs. gastric vs. gynecological) (*p* = 0.45) or by ascites status (ascites [+] vs. ascites [−]; *p* = 0.41). Pairwise Wilcoxon comparisons among the three tumor types also yielded no significant differences (*p* ≥ 0.34). Among the 17 patients with colorectal cancer, alpha diversity did not differ significantly across the four clinical stages (*p* = 0.43), and subsequent pairwise Wilcoxon comparisons were nonsignificant (*p* ≥ 0.13). Patients were further classified as metastatic (Meta) vs. non-metastatic (non-Meta); however, again, no significant difference was found in Shannon diversity (*p* = 0.13). Overall, these clinical stratifications—stage, metastatic status, tumor type, and ascites status—were not associated with statistically significant changes in urinary microbiome alpha diversity.

#### 3.4.2. Beta Diversity: Lack of Distinct Clusters Among Clinical Subgroups

Beta diversity was assessed using Bray–Curtis dissimilarity at the species level and visualized via PCoA. PERMANOVA was conducted to evaluate potential differences in microbial community composition across cancer type, clinical stage, metastatic status, and ascites status. Statistical ellipses were drawn only for groups with four or more samples (Figure S3). PC1 and PC2 captured 13.23% and 10.42% of the variation, respectively. PERMANOVA indicated no significant group differences (*p* = 0.4314). In patients with ascites (+) vs. those without (−), PC1 and PC2 explained 13.24% and 10.14% of the variation, respectively. PERMANOVA returned *p* = 0.9114, suggesting no distinct compositional divergence. PC1 and PC2 accounted for 15.31% and 12.94% of the total variation, respectively. PERMANOVA showed no significant difference among the four stage groups (*p* = 0.8368). When comparing Meta vs. non-Meta, PC1 explained 15.31% of the variation, and PC2 explained 12.94%. PERMANOVA yielded *p* = 0.3607, indicating no significant separation.

### 3.5. Flow Cytometric Analysis Reveals an Immunosuppressive Profile in Malignant Ascites

**Figure 5:**
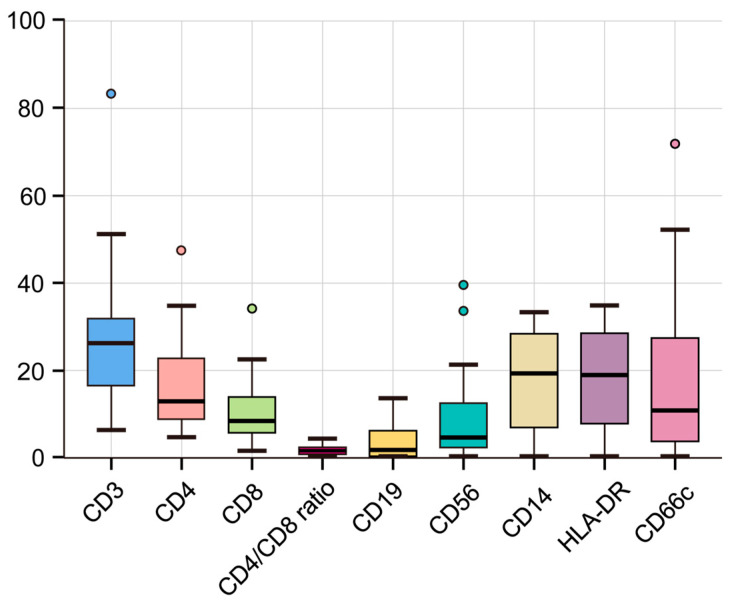
Flow cytometric analysis of immune cell populations in malignant ascitic fluid. Box plots show the proportions of various immune subsets in ascitic fluid (percentage of total cells), including CD3+ (T cells), CD4+ (T helper cells), CD8+ (cytotoxic T cells), the CD4+/CD8+ ratio, CD19+ (B cells), CD56+ (NK cells), HLA-DR (MHC class II), and CD66c+ (granulocytes). Each box extends from the first to the third quartile, with the horizontal line indicating the median.

Ascitic fluid samples from 15 of the 20 patients with ascites underwent further flow cytometric evaluation to characterize immune cell populations (Table 3). The mean age of these 15 patients was 61.87 ± 12.74 years (range: 35–82), and seven (46.7%) were male. Median (IQR) values for key immune markers in the ascitic fluid were as follows: CD4/CD8 ratio, 1.63 (0.98–2.40); CD19, 2.23 (0.52–6.10); CD56, 4.64 (3.0–11.13); HLA-DR, 18.86 (10.38–26.74); and CD66c, 11.07 (4.0–26.55). Figure 5 presents a box-and-whisker plot illustrating the distribution of major immune subsets (e.g., CD3+, CD4+, CD8+, and so on) across the 15 ascitic fluid samples. Notably, the CD4/CD8 ratio showed considerable interpatient variability, spanning from below 1.0 up to nearly 2.4, which may reflect heterogeneity in T-cell subset dynamics. The frequencies of B cells (CD19+) and NK cells (CD56+) were relatively low overall but varied widely among individuals. Meanwhile, HLA-DR (an MHC class II molecule) and CD66c (commonly expressed on granulocytes) also displayed broad ranges, suggesting potential differences in antigen-presenting capability and granulocyte involvement in malignant ascites.

## 4. Discussion

Our study indicates that malignant ascites is largely sterile, as 16S rRNA sequencing failed to detect meaningful bacterial communities in most samples. This observation aligns with prior reports showing minimal bacterial loads in cirrhotic ascites and spontaneous bacterial peritonitis (SBP) when assessed using high-sensitivity techniques [9].

While gut and bladder microbiome profiles did not differ significantly based on the presence of ascites, peritoneal metastasis correlated with changes in certain taxa (e.g., Clostridia, Gammaproteobacteria). Specifically, Clostridia and Gammaproteobacteria were relatively enriched in the metastatic group, whereas Bacilli predominated in non-metastatic patients. Within the Bacilli class, Lactobacillus is recognized as a beneficial gut bacterium that modulates inflammation and bolsters anti-tumor immunity in various cancers [16]. By contrast, Gammaproteobacteria has been linked to reduced chemotherapy efficacy (e.g., gemcitabine) and proposed as a potential biomarker for colorectal and pancreatic cancers [17,18]. Moreover, many Clostridia produce short-chain fatty acids (SCFAs) that protect the intestinal mucosa and exhibit anti-inflammatory effects, yet pathogenic species such as *Clostridioides difficile* also fall under this taxonomic class [19,20]. This functional heterogeneity complicates class-level interpretations.

In our subgroup analyses, the only finding that reached statistical significance was the increase in gut alpha diversity among stage IV colorectal cancer patients compared with stage I (*p* = 0.04). This observation contrasts with many studies that associate advanced cancer with reduced diversity and could be explained by the transient expansion of opportunistic microbes under compromised conditions (e.g., altered diet, immune dysfunction). Interestingly, the bladder microbiome exhibited an opposite trend—disease progression tended to coincide with lower alpha diversity—suggesting that the gut and bladder niches may respond differently to tumor burden or systemic factors.

However, other observed patterns, such as the subtle shift in beta diversity (PCoA) in patients with peritoneal metastases, did not reach statistical significance (PERMANOVA *p* > 0.05) and thus remain hypothesis-generating rather than conclusive. It is possible that the small sample size or heterogeneous prior treatments (e.g., chemotherapy, immunotherapy) masked underlying differences.

Flow cytometry of ascitic fluid showed lower proportions of T cells (CD3+, CD4+, CD8+) and NK cells (CD56+) compared with historical data from cirrhotic ascites, suggesting an immunosuppressive environment potentially driven by high tumor burden and elevated cytokine levels (Figure S4) [21,22]. Notably, the large interpatient variability in CD4/CD8 ratios and NK cell frequencies underscores the complexity of tumor–immune interactions, which may differ by cancer subtype, disease stage, and individual host factors. These observations indicate that malignant ascites represents more than a simple by-product of tumor growth; it is a dynamic microenvironment where immunosuppressive cells (e.g., myeloid-derived suppressor cells (MDSCs)), regulatory T cells (Tregs), and factors (e.g., TGF-β, IL-10) collectively undermine anti-tumor immunity by promoting T-cell dysfunction and reducing antigen presentation [23]. Although immune checkpoint inhibitors have shown promise in advanced cancers, the multifaceted nature of immunosuppression in malignant ascites indicates that additional strategies (e.g., targeting MDSCs/Tregs) could be explored, given the immunosuppressive features observed in malignant ascites, though further validation is needed.

Although numerous studies have examined how the microbiome influences cancer development, few have addressed the interplay among the peritoneum, gut, and bladder in malignant ascites. Existing research on a “peritoneum–gut axis” or “peritoneum–bladder axis” has primarily focused on chronic kidney disease patients receiving dialysis or on inflammatory conditions such as endometriosis and urinary tract infections [24]. Our study appears to be among the first to investigate malignant ascites and peritoneal metastasis in conjunction with both gut and bladder microbiomes.

Despite these contributions, several limitations warrant attention. First, the relatively small sample size reduced statistical power in subgroup analyses, potentially obscuring significant patterns—particularly in LEfSe results. Second, multiple comparisons across various taxonomic levels could inflate the risk of type I errors, demanding a cautious interpretation of borderline *p*-values and LDA scores. Third, 16S rRNA-based methods in low-biomass samples like ascitic fluid can underestimate microbial communities if rare taxa remain below the detection threshold or if contamination biases occur. Fourth, our cross-sectional design provided only a single time point, preventing an assessment of longitudinal changes in microbiota or immune profiles throughout disease progression. Lastly, confounders such as antibiotic use, nutritional status, and tumor burden were not fully controlled and may have influenced the observed microbial patterns.

Furthermore, the following key unmet needs remain: while our findings highlight a largely sterile malignant ascitic fluid, the clinical significance of specific taxa in the gut and bladder, especially Clostridia, Gammaproteobacteria, and Bacilli, warrants deeper investigation. Comprehensive metagenomic and metabolomic approaches could elucidate functional pathways, such as SCFA production or immunomodulatory processes, which might be pivotal in malignant ascites development and progression. A more nuanced understanding of the peritoneum–gut and peritoneum–bladder axes could inform targeted interventions aimed at modulating these microbiomes to improve immune responses or therapeutic efficacy. Future studies should adopt longitudinal designs to capture dynamic shifts in microbial communities and immune profiles over the course of disease and treatment while simultaneously controlling for confounding variables (e.g., antibiotic use, nutritional alterations).

## 5. Conclusions

In conclusion, malignant ascites harbors a minimal bacterial load alongside an immunosuppressive environment marked by decreased T-cell and NK cell proportions. Nevertheless, the relative enrichment of Clostridia and Gammaproteobacteria in peritoneal metastases—and higher Bacilli levels in non-metastatic cases—implies that microbial factors may influence or reflect tumor behavior within the peritoneal cavity. Given the limitations of 16S rRNA analysis in low-biomass samples, metagenomic or metatranscriptomic approaches might better capture rare taxa or microbial gene expression. Metabolomic studies could also reveal key microbial byproducts (e.g., short-chain fatty acids, secondary bile acids) that shape immune activity and tumor progression. Larger-scale, longitudinal studies are warranted to validate our observations and determine whether microbiota modulation can reshape the tumor–immune milieu in malignant ascites. Ultimately, understanding how gut and bladder microbial communities integrate with the peritoneal environment may open avenues for novel diagnostic markers and therapeutic strategies in advanced malignancies.

## Figures and Tables

**Table 1:** Demographic and clinical characteristics of the study population.

Characteristic	n = 66
**Age, years**	
Mean ± SD (range)	64.79 ± 10.84 (31–87)
**Sex**	
Male	33 (50.0%)
Female	33 (50.0%)
**Solid malignancies**	
Colorectal cancer	48 (72.7%)
Gastric cancer	6 (9.1%)
Ovary cancer	10 (15.2%)
Others	2 (3.0%)
**Stage**	
I/II	18 (27.3%)
III	19 (28.8%)
IV	29 (43.9%)
**Group**	
With ascites	20 (30.3%)
Without ascites	46 (69.7%)
**Peritoneal metastases**	27 (40.9%)
Pathologic confirmed	12 (60.0%)
Atypical cell	6 (30.0%)
Pathological negative	2 (10.0%)

**Table 2:** Baseline characteristics of malignant ascites samples.

No.	Sex	Age	Cancer Type	Ascites 16sR	Ascites Culture	Ascites WBC	Ascites PMN	Ascites CEA
1	M	72	Urachal cancer	Too low bac load		648	45	4438
2	F	68	Ovary cancer	Too low bac load		6768	5617	495,370
3	F	53	Ovary cancer	Too low bac load		1040	21	0.33
4	F	78	Endometrial cancer	Too low bac load		140	1	0.36
5	F	65	Colon cancer	Enterococcus (50.9%), Bacteroides (18.1%)	Enterococcus faecalis	680	265	5896.0
6	F	56	Cervical cancer	Too low bac load		290	0	NA
7	M	35	AGC	Too low bac load		3300	0	2049
8	F	65	Primary peritoneal cancer	Too low bac load		1120	22	2.42
9	F	82	Extrapulmonary NET	Too low bac load		1080	259	0.86
10	M	81	Cecal cancer	Too low bac load		900	18	4954
11	F	70	Ovary cancer	Too low bac load		396	4	1.35
12	F	46	Colon cancer	Too low bac load		3600	3348	27.3
13	F	58	Colon cancer	Too low bac load		190	6	217
14	M	63	Appendiceal cancer	Too low bac load		980	59	114
15	M	62	Colon cancer	Too low bac load		310	0	105
16	M	65	AGC	Too low bac load		360	4	3937
17	M	70	AGC	Too low bac load		3816	2519	271
18	F	57	AGC	Too low bac load		8	0	68.5
19	F	59	AGC	Too low bac load		48	0	1268
20	M	56	AGC	Too low bac load		369	4	301

**Table 3:** Flow cytometric analysis of malignant ascites.

	n = 15
**Age, years**	
Mean ± SD (range)	61.87 ± 12.74 (35–82)
**Sex**	
Male	7 (46.67%)
Female	8 (53.33%)
**Flow cytometry**	Median (IQR)
CD3	26.03 (17.21, 31.67)
CD4	12.81 (9.54, 21.39)
CD8	8.37 (5.87, 13.12)
CD4/CD8 ratio	1.63 (0.98, 2.4)
CD19	2.23 (0.52, 6.1)
CD56	4.64 (3, 11.13)
HLA-DR	18.86 (10.38, 26.74)
CD66c	11.07 (4, 26.55)

## Data Availability

The datasets generated and/or analyzed in this study are available from the corresponding author upon reasonable request.

## Supplementary Materials

The following supporting information can be downloaded at: https://www.mdpi.com/article/10.3390/cancers17081280/s1, Figure S1: Principal coordinates analysis (PCoA) of the gut microbiome (Bray–Curtis); Figure S2: Taxa abundance bar plots paired with Lefse outputs of Figure 3; Figure S3: PCoA of the urine microbiome (Bray–Curtis) across clinical subgroups; Figure S4: Ascites lymphocytic surface markers of cirrhotic patients without ascites infections.

## Abbreviations

The following abbreviations are used in this manuscript:

RAAS	renin–angiotensin–aldosterone system
VEGF	vascular endothelial growth factor
IL-2	interleukin-2
TNF-alpha	tumor necrosis factor-alpha
EMT	epithelial–mesenchymal transition
HLA-DR	human leukocyte antigen-DR
SBP	spontaneous bacterial peritonitis
LDA	linear discriminant analysis
LEfSe	linear discriminant analysis effect size
PCoA	principal coordinates analysis
PERMANOVA	permutational multivariate analysis of variance
NK	natural killer
SCFA	short-chain fatty acid
MHC	major histocompatibility complex
