# Supplementary material for: Microbial and Immune Landscape of Malignant Ascites: Insights from Gut, Bladder, and Ascitic Fluid Analyses
Source: Cancers (Basel). 2025 Apr 10;17(8):1280. doi: 10.3390/cancers17081280 (PMC12025743; doi:10.3390/cancers17081280)
Supplement: Supplementary file 1 [file cancers-17-01280-s001.zip › cancers-3541198-supplementary.pdf]

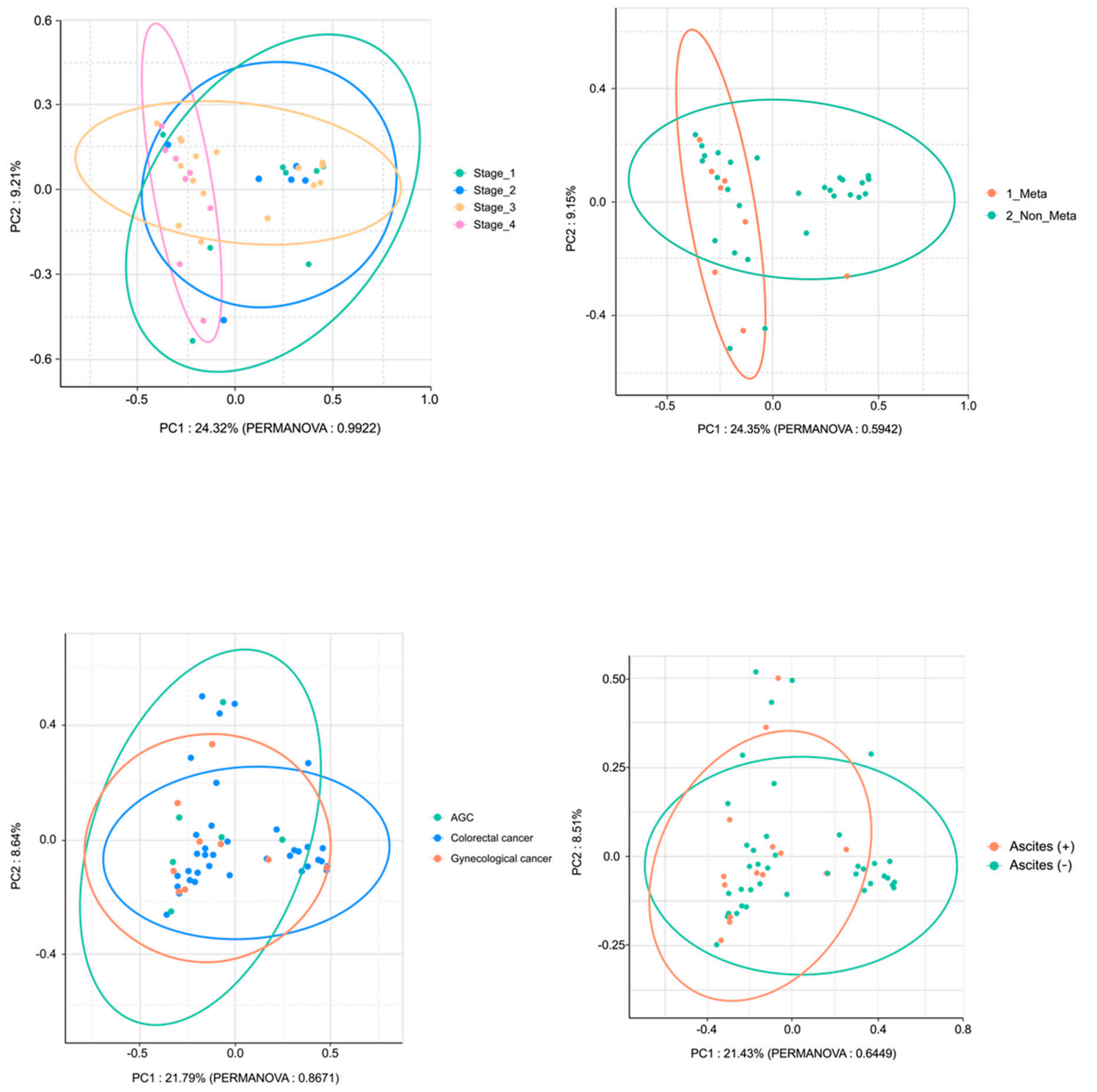

Supplementary Figure 1. Principal coordinates analysis (PCoA) of the gut microbiome (Bray–Curtis). PCoA plots show overall gut microbial composition by (top left) cancer stage (I–IV), (top right) peritoneal metastasis status (Meta vs. non_Meta), (bottom left) cancer type (gastric, colorectal, gynecological), and (bottom right) ascites presence (Ascites+ vs. Ascites−). 95% confidence ellipses are drawn for groups with ≥4 samples. PERMANOVA p-values are shown in each panel.

(A)

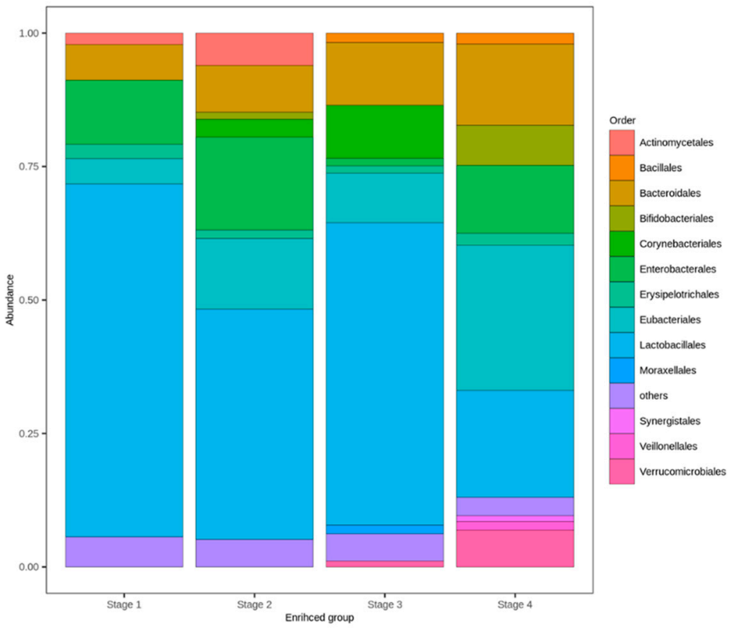
1.00
0.75
0.50
0.25
0.00
Abundance
Stage 1
Stage 2
Stage 3
Stage 4
Enrihced group
Order
Actinomycetales
Bacillales
Bacteroidales
Bifidobacteriales
Corynebacteriales
Enterobacterales
Erysipelotrichales
Eubacteriales
Lactobacillales
Moraxellales
others
Synergistales
Veillonellales
Verrucomicrobiales

(B)

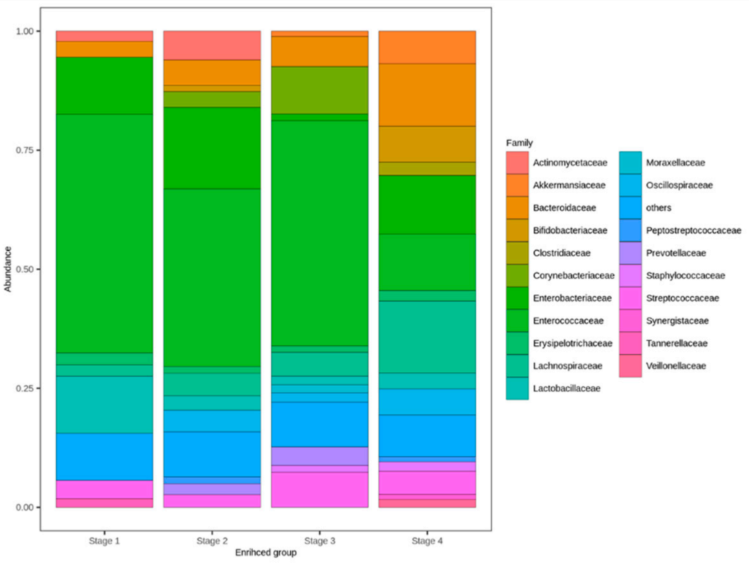
1.00
0.75
0.50
0.25
0.00
Abundance
Stage 1
Stage 2
Stage 3
Stage 4
Enrihced group
Family
Actinomycetaceae
Akkermansiaceae
Bacteroidaceae
Bifidobacteriaceae
Clostridiaceae
Corynebacteriaceae
Enterobacteriaceae
Enterococcaceae
Erysipelotrichaceae
Lachnospiraceae
Lactobacillaceae
Moraxellaceae
Oscillospiraceae
others
Peptostreptococcaceae
Prevotellaceae
Staphylococcaceae
Streptococcaceae
Synergistaceae
Tannerellaceae
Veillonellaceae

(C)

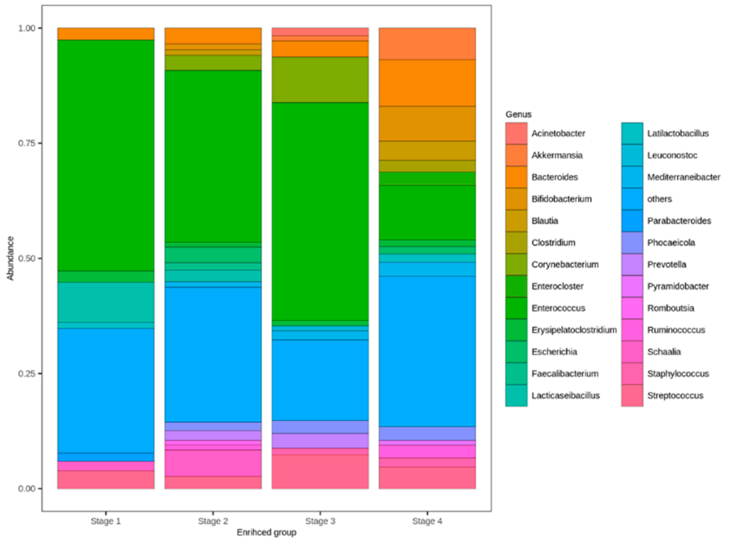
1.00
0.75
0.50
0.25
0.00
Abundance
Stage 1
Stage 2
Stage 3
Stage 4
Enrihced group
Genus
Acinetobacter
Akkermansia
Bacteroides
Bifidobacterium
Blautia
Clostridium
Corynebacterium
Enterocloster
Enterococcus
Erysipelatoclostridium
Escherichia
Faecalibacterium
Lacticaseibacillus
Latilactobacillus
Leuconostoc
Mediterraneibacter
others
Parabacteroides
Phocaeicola
Prevotella
Pyramidobacter
Romboutsia
Ruminococcus
Schaalia
Staphylococcus
Streptococcus

(D)

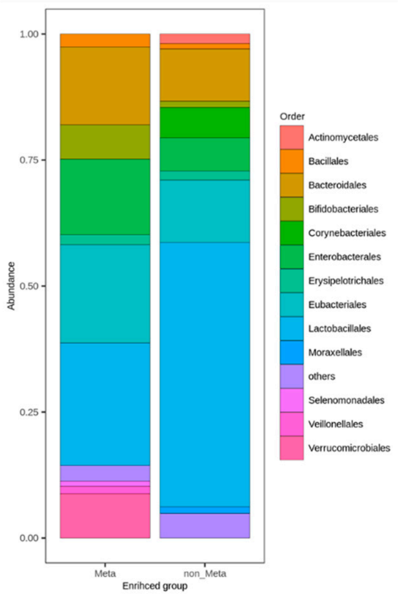
1.00
0.75
0.50
0.25
0.00
Abundance
Meta
non_Meta
Enrihced group
Order
Actinomycetales
Bacillales
Bacteroidales
Bifidobacteriales
Corynebacteriales
Enterobacterales
Erysipelotrichales
Eubacteriales
Lactobacillales
Moraxellales
others
Selenomonadales
Veillonellales
Verrucomicrobiales

(E)

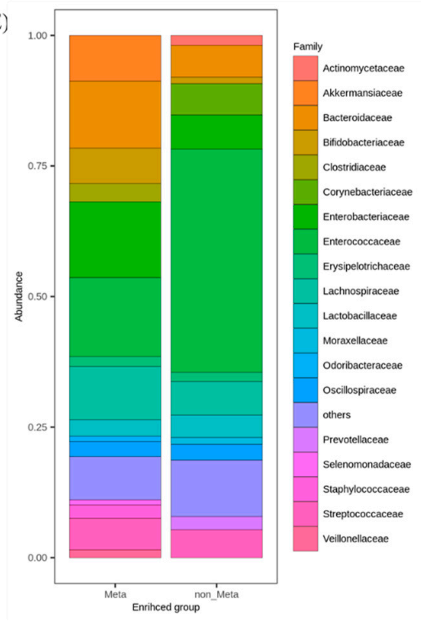
1.00
0.75
0.50
0.25
0.00
Abundance
Meta
non_Meta
Enrihced group
Family
Actinomycetaceae
Akkermansiaceae
Bacteroidaceae
Bifidobacteriaceae
Clostridiaceae
Corynebacteriaceae
Enterobacteriaceae
Enterococcaceae
Erysipelotrichaceae
Lachnospiraceae
Lactobacillaceae
Moraxellaceae
Odoribacteraceae
Oscillospiraceae
others
Prevotellaceae
Selenomonadaceae
Staphylococcaceae
Streptococcaceae
Veillonellaceae

(F)

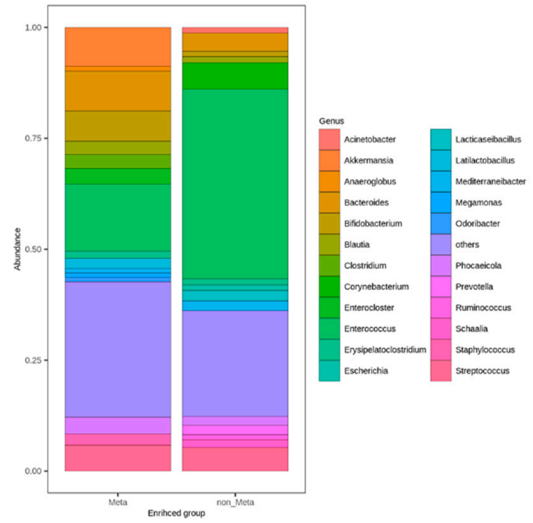
1.00
0.75
0.50
0.25
0.00
Abundance
Meta
non_Meta
Enrihced group
Genus
Acinetobacter
Akkermansia
Anaeroglobus
Bacteroides
Bifidobacterium
Blautia
Clostridium
Corynebacterium
Enterocloster
Enterococcus
Erysipelatoclostridium
Escherichia
Lacticaseibacillus
Latilactobacillus
Mediterraneibacter
Megamonas
Odoribacter
others
Phocaeicola
Prevotella
Ruminococcus
Schaalia
Staphylococcus
Streptococcus

Supplementary Figure 2. Taxa abundance bar plots paired with Lefse outputs of figure 3. (A)~(C) are abundance bar plots of Order, Family, and Genus level, respectively, according to the cancer stage and (D)~(F) are abundance bar plots of Order, Family, and Genus level, respectively, according to the mestastases.

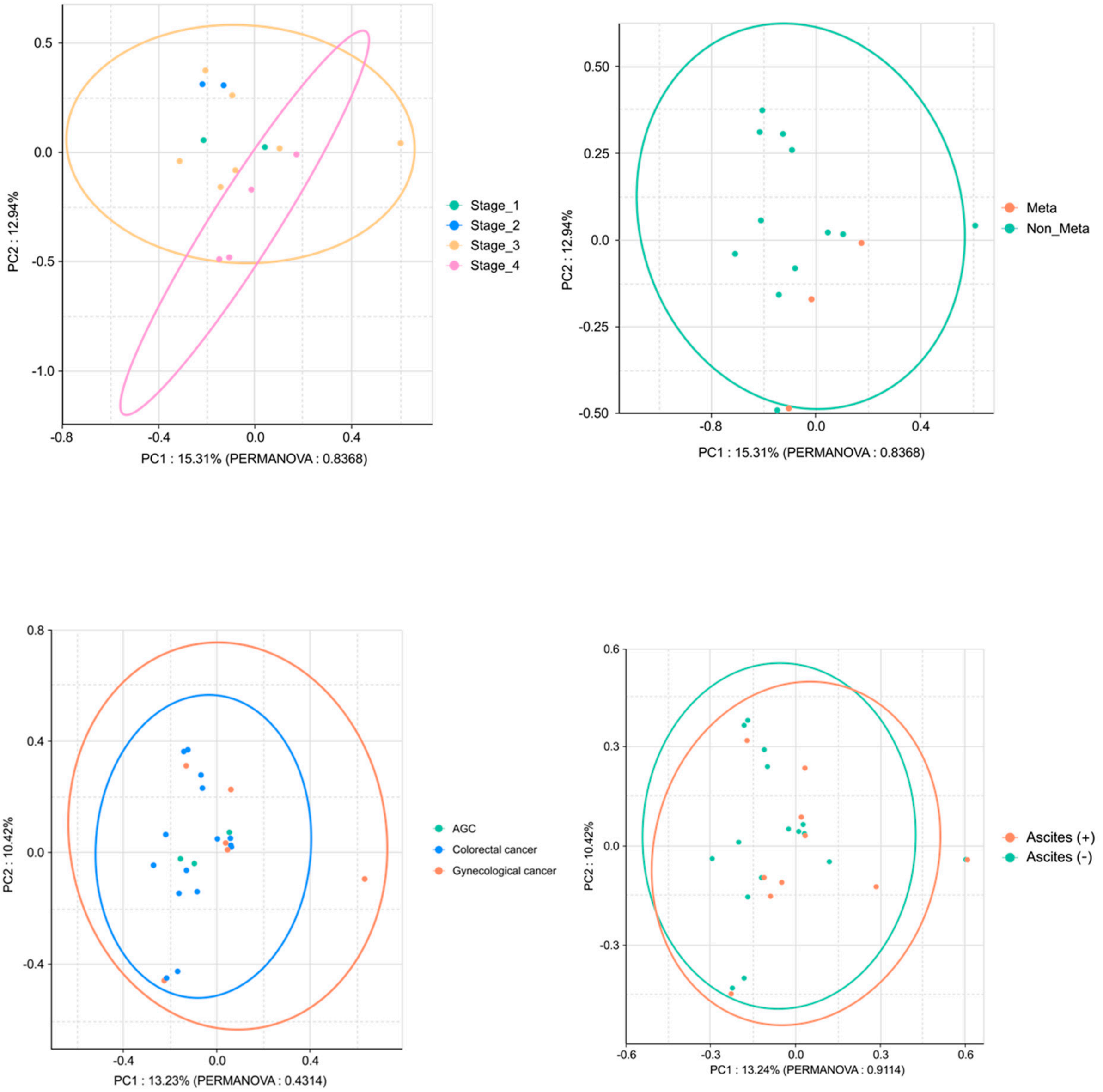

Supplementary Figure 3. PCoA of the urine microbiome (Bray–Curtis) across clinical subgroups. Each panel displays the differences in the urinary microbiome by (top left) clinical stage (I–IV), (top right) peritoneal metastasis (Meta vs. non_Meta), (bottom left) cancer type (gastric, colorectal, gynecological), and (bottom right) ascites status (Ascites+ vs. Ascites−). Where possible ($n \geq 4$), 95% confidence ellipses were drawn. PERMANOVA results (p-values) are indicated in each plot.

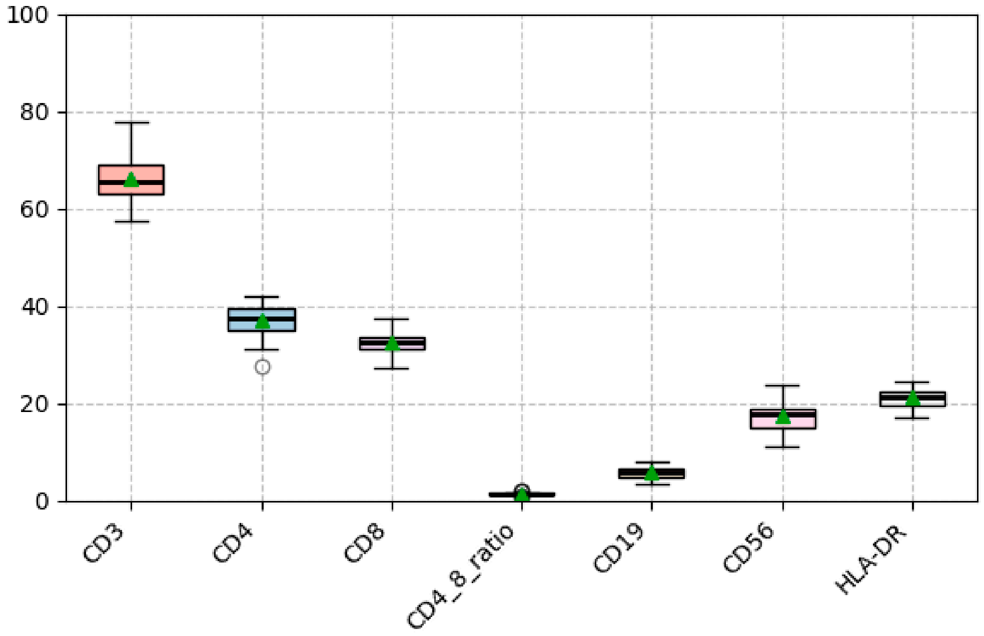

Lymphocyte cellular surface markers (%)	mean ± SD
CD3	66.28 ± 4.33
CD4	36.84 ± 3.29
CD8	32.57 ± 2.04
CD4_8_ratio	1.43 ± 0.25
CD19	5.91 ± 1.21
CD16 + 56	18.24 ± 3.77
HLA-DR	20.73 ± 2.26

Supplementary Figure 4.

Ascites lymphocytic surface markers of cirrhotic patients without ascites infections. Adapted from Kiyici et al. [21], with permission from John Wiley & Sons, License Number 5995850128711**.**

Box plots illustrate the proportions of T cells (CD3+, CD4+, CD8+), B cells (CD19+), NK cells (CD56+), and granulocytes (CD66c+), as well as MHC class II expression (HLA-DR). These data serve as a reference baseline, enabling direct comparison with the malignant ascites flow cytometry findings in Figure 5. Note that cirrhotic ascites typically exhibits higher T/NK cell proportions, whereas malignant ascites shows a more immunosuppressive profile overall (see Discussion for details).
